# Randomized clinical trial comparing lumbar percutaneous hydrodiscectomy with lumbar open microdiscectomy for the treatment of lumbar disc protrusions and herniations

**DOI:** 10.6061/clinics/2016(05)06

**Published:** 2016-05

**Authors:** Alexandre Fogaça Cristante, Ivan Diasda Rocha, Raphael Martus Marcon, Tarcísio Eloy Pessoa de Barros Filho

**Affiliations:** Instituto de Ortopedia e Traumatologia do Hospital das Clínicas da Faculdade de Medicina da Universidade de São Paulo (IOT-HCFMUSP), Departamento Ortopedia e Traumatologia, Divisão de Cirurgia de Coluna Vertebral, São Paulo/SP, Brazil

**Keywords:** Discectomy, Percutaneous Discectomy, Low Back Pain, Spine

## Abstract

**OBJECTIVES:**

Hydrodiscectomy is a new technique used for percutaneous spinal discectomy that employs a high-intensity stream of water for herniated disc ablation and tissue aspiration. No previous clinical study has examined the effects of percutaneous hydrodiscectomy. The aim of this study is to evaluate the outcomes of hydrodiscectomy compared to open microdiscectomy regarding pain, function, satisfaction, complications and recurrence rates.

**METHODS:**

In this randomized clinical trial, patients referred to our tertiary hospital for lumbar back pain were recruited and included in the study if they had disc protrusion or small herniation in only one level, without neurological deficits and with no resolution after six weeks of conservative treatment. One group underwent open microdiscectomy, and the other group underwent percutaneous microdiscectomy via hydrosurgery. Function was evaluated using the Oswestry Disability Index and pain was assessed using a visual analog scale. Evaluations were performed preoperatively, and then during the first week and at one, three, six and twelve months postoperatively. Personal satisfaction was verified. Clinicaltrials.gov: NCT01367860.

**RESULTS:**

During the study period, 20 patients were included in each arm and 39 completed one-year of follow-up (one patient died of unrelated causes). Both groups exhibited equal improvement on the visual analog scale and Oswestry evaluations after treatment, without any significant differences. The improvement in the lumbar visual analog scale score was not significant in the hydrodiscectomy group (*p*=0.138). The rates of infection, pain, recurrence and satisfaction were similar between the two groups.

**CONCLUSION:**

Percutaneous hydrodiscectomy was demonstrated to be as effective as open microdiscectomy for reducing pain. The rates of complications and recurrence of herniation were similar between groups. Patient satisfaction with the treatment was also similar between groups.

## INTRODUCTION

Open surgical procedures to treat spinal herniations are associated with complications such as epidural hematoma, infection, residual instability, recurrence of disc herniation and post-laminectomy syndrome, which suggests a need to develop minimally invasive treatment techniques 1. Percutaneous lumbar discectomy was first described by Hijikata in 1989 2. It allowed partial resection of the nuclear substance via a posterolateral approach, leading to a reduction of the intradiscal pressure and relief of irritation of the nerve root or the pain receptors around the disc. As described by the author of that study, “The extraction of the herniated portion of the disc is not achieved by the procedure. However, the amount of herniated disc substance may be reduced by disc decompression with suction.” Many percutaneous discectomy techniques have been described since, with relative success in the treatment of pain and disability resulting from disc protrusions or small herniated discs associated with radiculopathy 3-9.

The reported success rate for percutaneous lumbar discectomy ranges from 29% to 96%, *versus* 72% to 90% for the microdiscectomy technique, depending on the evaluation method 10,11. The techniques used for percutaneous lumbar discectomy are heterogeneous with respect to the mechanism of action and the amount of disc removed, and include chemonucleolysis 12-14, automatic percutaneous discectomy 15,16, microendoscopic discectomy 17,18, endoscopic discectomy 19,20, decompression of the disc by laser 21,22, dynamic stabilization 23, electrothermal ablation and radiofrequency ablation 24,25. At present, controversy exists regarding the role of percutaneous discectomy for contained herniated discs; however, a recent study found that patients who underwent a percutaneous endoscopic lumbar discectomy for herniated discs had less blood loss, reduced tissue damage and a shorter hospital stay 1.

Hydrodiscectomy is a new technique introduced for percutaneous discectomy. It uses a focused, high-intensity stream of water for ablation, which aspirates the tissue at the same time. It resulted in a fast, precise procedure in a study using cadavers 26. However, no previous studies have been conducted that have examined the effects of percutaneous hydrodiscectomy in living humans.

The objective of this study was to evaluate the results of hydrodiscectomy and compare them to the gold standard procedure, open microdiscectomy, regarding pain, function, satisfaction with the surgery and complications or recurrence. The hypothesis examined was that the new technique was as safe and effective as traditional percutaneous surgery.

## MATERIALS AND METHODS

### Design, setting and ethics

This was a randomized, controlled, single blind study undertaken in a university at a public hospital in Brazil, comprising a convenience sample of consecutive patients admitted between June 2011 and January 2012, with follow-up until January 2013. The study was approved by the local Ethics Committee and was registered in ClinicalTrials.gov under protocol NCT01367860. It complied with the Declaration of Helsinki (1983). All patients provided signed informed consent.

### Eligibility criteria and allocations

Patients 18 to 76 years old who were referred to our tertiary hospital for the treatment of lumbar back pain were recruited. They were included in the study if they had the following: disc protrusion or small herniation at only one level, as demonstrated by magnetic resonance imaging (MRI); MRI findings compatible with the symptoms; no neurological deficits; and no resolution after six weeks of conservative treatment. The exclusion criteria were as follows: neurological changes observed during conservative treatment, pregnancy, a clinical status that was not adequate for surgical procedures and those who did not provide informed consent. After inclusion in the study, the patients were evaluated for pain and function and were then randomly allocated to one of two groups by block randomization; one group of patients received an open microdiscectomy and the other group underwent percutaneous microdiscectomy by hydrosurgery.

Randomization was performed in the operating room (OR) with opaque, sealed envelopes that were previously prepared in blocks of four each. Once the patient was admitted and prepared for surgery, one researcher (IDR) evaluated the participant for function and pain as described below. This evaluator was unaware of the treatment to be conducted and the patient undergoing the evaluations was also blind to the procedure. Then, the chief nurse (not an author) opened one envelope and disclosed the procedure to the surgeon. One experienced spine surgeon (AFC) performed all of the procedures in this study, with the help of assistants. The patient was blinded to the type of surgery before the procedure, although he/she could later observe the scar, which was larger and more centrally located in individuals who underwent open surgery.

### Interventions

Microdiscectomy was performed with the patient under general anesthesia in a knee-chest position with hip flexion and with the aid of magnifying glasses and fluoroscopy. We created a longitudinal central opening and performed dissection up to the lamina during the small laminectomy procedure, followed by a flavectomy, hemostasis, removal of roots and a discectomy.

Hydrodiscectomy was performed with the patient under sedation, which allowed the surgeon to observe the movement of the legs during the procedure. The patient was placed lying in prone position and fluoroscopy was used to locate the desired level for the operation. Blocking was performed with lidocaine (5 ml, 1%). A needle was inserted percutaneously and posterolaterally, via an extrapedicular approach, below the neural foramen in the center of the disc using the traditional approach for a discectomy. Front and profile fluoroscopy were used to confirm the desired position. A dilator was introduced into the disc, followed by placement of a cannula over the dilator and into the outer annulus. The cannula was then advanced into the inner annulus. The dilator and needle were removed and the device for hydrosurgery (SpineJet Hydrosurgery System, Hydrocision, North Billerica, MA, USA) was introduced via the cannula access. Manual light pistoning and rotation movements were performed for approximately three to five minutes. The disc material was removed by suction.

All patients underwent the same physical therapy protocol in our hospital after surgery. The physical therapists were blinded to the surgical procedure and were informed only that the patient had undergone herniated disc ablation for the treatment of low back pain.

### Outcomes and statistical analysis

Function was evaluated using the Oswestry Disability Index (ODI) 27 and pain was evaluated using a visual analog scale (VAS) 28 for lumbar pain and pain radiating to the legs. Function and pain were the main outcomes analyzed. These evaluations occurred preoperatively, during the first week after surgery and at one, three, six and twelve months postoperatively. The same researcher responsible for the ODI and VAS evaluations investigated the patients' satisfaction with the treatment. Personal satisfaction with the surgical results was investigated by asking the patient if he/she was satisfied to the point that they would undergo such a treatment again, with an affirmative answer indicating that the procedure alleviated pain and the patient considered it valuable.

Surgical failure was considered to have occurred when radiating pain that was equal to or more intense than that experienced preoperatively was present at 30 days post-operatively. In these cases, an MRI examination was performed to evaluate the patient to determine whether they had indications for surgical revision with microdiscectomy, allowing proper planning for the removal of herniated material.

The statistical analysis was performed based on an intention to treat (ITT) method. Normality was examined using the Kolmogorov-Smirnov test, and if confirmed, data were analyzed with a t-test. All normally distributed continuous data were analyzed using unpaired t-tests and expressed as the means and standard deviations. Baseline categorical data were presented as proportions between groups and statistically tested with the chi-square test and, when necessary, with Fisher's exact test. Statistical significance was established at a value of *p*<0.05. The statistical analysis was performed with the Statistical Package for Social Sciences (SPSS) software, version 19.0 for Windows.

## RESULTS

During the study period, 40 patients were included in the study, 20 in each arm. Except for one patient in the hydrosurgery group who died from other causes in the sixth month after surgery, all patients completed one-year of follow up. The demographic and clinical baseline data are shown in Table 1. The only difference between the groups at the end of follow-up was the result of the ODI, which was significantly better in the group of patients who underwent a hydrodiscectomy.

**Figure 1:**
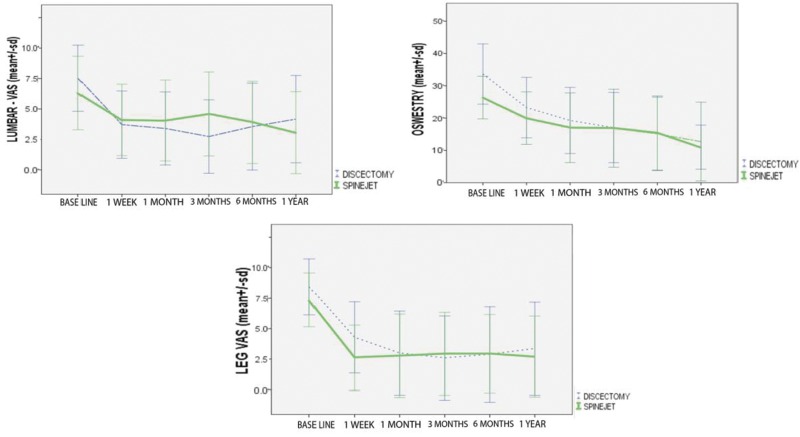
Outcomes during follow up.

As shown in Table 2 and Figure 1, both groups exhibited equal improvements in the VAS (lumbar and leg pain) and functional evaluations (ODI) after treatment, without any significant differences between them. From baseline until the evaluation performed during the first week, the improvements in the VAS score for leg pain and the functional evaluation (ODI) were significantly different for both groups (*p*<0.001 for both scales) and significant differences were found for the VAS score for lumbar pain in patients who underwent open discectomy (*p*<0.001). The lumbar VAS score was not significantly improved (*p*=0.138) for the group of patients who underwent hydrodiscectomy.

In the open discectomy group, one case of superficial infection occurred, which was treated with antibiotics; three cases of residual pain were observed, in which a postoperative MRI investigation showed proper removal of the herniation but with residual epidural fibrosis (revision surgery was not indicated in these cases); and two cases of recurrence were observed, with revision indicated in both cases. In the group of patients who underwent hydrodiscectomy, one case of infection occurred, which was treated with antibiotics. In four patients, the pain did not improve after treatment, and these patients all underwent open microdiscectomy two months after the first procedure, with subsequent improvement of their pain. One patient in this group was HIV-positive and died six months after treatment due to pneumonia. No significant difference was observed between the groups regarding the frequency of infection (*p*>0.05), residual pain or recurrence (according to Fisher's exact test and the chi-squared test).

In the hydrosurgery group, 13 patients (68.5% of those who completed the follow-up) were satisfied with the treatment compared to 17 patients (85%) in the open discectomy group, resulting in no significant difference between the groups according to a chi-squared test (*p*=0.27).

## DISCUSSION

Several randomized controlled trials have compared percutaneous discectomy with microdiscectomy 29. In a randomized study including patients with small disc herniation or contained herniated discs, percutaneous endoscopic discectomy was shown to produce results comparable to open microdiscectomy, despite removing smaller amounts of disc material (4.3±1.3 g compared to 12.8±3 g) 7. However, other randomized studies of patients with only herniated discs have demonstrated that microdiscectomy was superior to automated percutaneous discectomy, with varying amounts of disc material removed using the percutaneous technique 10,15.

Some devices for percutaneous discectomy have been criticized because they cannot remove the posterior portion of the nuclear material of the herniated disc 11. Some methods remove very small amounts of tissue, with little change in the volume 30, and the clinical improvement after percutaneous discectomy is more likely to be due to the reduced intradiscal pressure and volume than the direct removal of the herniated tissue in many situations 31. However, studies with cadavers using hydrosurgery or similar methods (such as “shavers”) have shown macroscopic changes in the disc with the removal of a predictable and significant amount of material 26,32,33. No clinical study of hydrodiscectomy has been published in any indexed journal to date.

In our randomized clinical trial, significant improvements were observed in all outcome measures except the lumbar VAS score in the group of patients who underwent hydrodiscectomy. One possible explanation for this lack of significance, despite the trend toward improvement shown in Figure 1, could be the high variability of the results, which could have resulted in a type 2 error. Increasing the number of patients studied would help decrease this error. However, this is the first randomized clinical study on the subject, and it included only 20 subjects per arm.

In a randomized study similar to the present study, Chatterjee et al.^15^ compared the results of percutaneous microdiscectomy with those of automatic discectomy and observed a satisfaction rate of 80% for microdiscectomy compared to a rate of only 29% for automated percutaneous discectomy. In our study, we observed satisfaction rates of 85% for microdiscectomy and 69% for percutaneous hydrodiscectomy, which were not significantly different. Hydrodiscectomy is a percutaneous procedure that is performed as an outpatient surgery, which we found had similar rates of infections, residual pain and recurrence to the open procedure. Open microdiscectomy requires general anesthesia, a one to two day hospital stay and leaves a larger scar.

Improvements in pain were observed in the patients treated in both groups and the use of percutaneous hydrodiscectomy was shown to be as effective as the conventional open discectomy procedure. The complication rates in both groups were similar and limited to cases of infection. The rates of recurrent herniation were also similar between the groups, as were the rates of satisfaction with the treatment.

## AUTHOR CONTRIBUTIONS

Cristante AF designed the study, collected and interpreted data, wrote the manuscript and approved the final version of the manuscript. Rocha ID and Marcon RM collected and interpreted the data, helped with manuscript writing and critical revision and approved the final version of the manuscript. de Barros TE designed the study, helped with data interpretation, critically revised the manuscript and approved the final version of the manuscript. All authors are accountable for all aspects of the work regarding the accuracy and integrity of the study data.

## Figures and Tables

**Table 1:** Demographic and baseline clinical data.

Baseline variable	Discectomy (n=20)	Hydrodiscectomy (n=20)	*p*-value	Test
Age in years (mean±SD)	41.2±9.3	44.9±9.4	>0.05	Kolmogorov-Smirnov + t-test
Sex (female, %)	50	50	>0.05	chi-squared
Race (white, %)	85	65	>0.05	chi-squared
Education (>8 years, %)	50	50	>0.05	chi-squared
Married (yes, %)	90	65	>0.05	chi-squared
Manual labor (yes, %)	35	40	>0.05	chi-squared
Opioid use (yes, %)	35	45	>0.05	chi-squared
Currently working (no, %)	70	50	>0.05	chi-squared
Receiving government benefits (yes, %)	35	50	>0.05	chi-squared
Months away from work (mean±SD)	4.65±6.52	8.67±14.4	>0.05	Kolmogorov-Smirnov + t-test
Months of experiencing pain (mean ± SD)	16.25±20.2	33.5±47.3	>0.05	Kolmogorov-Smirnov + t-test
VAS score for lumbar pain preoperatively (mean±SD)	7.52±2.7	6.3±3	>0.05	Kolmogorov-Smirnov + t-test
VAS score for leg pain preoperatively (mean±SD)	8.42±2.3	7.36±2.2	>0.05	Kolmogorov-Smirnov + t-test
Preoperatively Oswestry score (mean±SD)	33.65±9.33	26.35±6.6	0.007	Kolmogorov-Smirnov + t-test

SD = standard deviation; VAS = visual analogue scale

**Table 2:** Visual analogue scale and Oswestry scale data (mean±standard deviation) during follow-up.

Outcomes	Discectomy (n=20)	Hydrodiscectomy (n=20)	*p*-value
VAS score for lumbar pain 1 week after the operation	3.71±2.76	4.1±2.93	> 0.05
VAS score for leg pain at 1 week	4.3±2.92	2.61±2.67	
Oswestry index at 1 week	23.25±9.37	20±8.13	
VAS score for lumbar pain 1 month after the operation	3.34±2.92	4.06±3.31	
VAS score for leg pain at 1 month	2.96±3.4	2.76±3.41	
Oswestry index at 1 month	19.25±10.23	10.78±2.41	
VAS score for lumbar pain 3 months after the operation	2.63±2.9	4.53±3.47	
VAS score for leg pain at 3 months	2.58±3.4	2.92±3.4	
Oswestry index at 3 months	17.05±10.9	16.85±12	
VAS score for lumbar pain 6 months after the operation	3.5±3.54	3.79±3.34	
VAS score for leg pain at 6 months	2.84±3.82	2.88±3.14	
Oswestry index at 6 months	15.37±11.45	15.1±11.37	
VAS score for lumbar pain 12 months after the operation	4.06±3.54	3.03±3.32	
VAS score for leg pain at 12 months	3.37±3.8	2.67±3.3	
Oswestry index at 12 months	11±6.82	12.7±12.2	
